# Diagnosis of Osteoarticular Tuberculosis: Perceptions, Protocols, Practices, and Priorities in the Endemic and Non-Endemic Areas of the World—A WAIOT View

**DOI:** 10.3390/microorganisms8091312

**Published:** 2020-08-28

**Authors:** Vikas M. Agashe, Ashok N. Johari, Mudit Shah, Rashid Anjum, Carlo Romano, Lorenzo Drago, Hemant K. Sharma, Thami Benzakour

**Affiliations:** 1 PD Hinduja Hospital, Mahim, Mumbai 400016, India; agashefam@gmail.com; 2 ENABLE International Centre for Paediatric Musculoskeletal Care, Mumbai 400016, India; drashokjohari@hotmail.com; 3 Dr. Agashe’s Maternity and Surgical Nursing Home, 116 Bazar ward, MN, Road Kurla, Mumbai 400070, India; muditshah160790@gmail.com; 4 Department of Orthopaedics, ASCOMS and Hospital, J and K, Jammu 180017, India; 5 Studio Medico Associato Cecca-Romanò, 20121 Milano, Italy; carlo.romano@unimi.it; 6 Clinical Microbiology, University of Milan, 20100 Milan, Italy; lorenzo.drago@unimi.it; 7 Hull University Teaching Hospitals, Anlaby Road, Hull HU3 2JZ, UK; h.sharma@hull.ac.uk; 8 Zerktouni Orthopaedic Clinic, Casablanca 20000, Morocco; t.benzakour@gmail.com

**Keywords:** osteoarticular tuberculosis, endemic, clinico-radiological diagnosis, GeneXpert, culture, drug sensitivity, drug-resistant tuberculosis

## Abstract

There has been a major resurgence of tuberculosis (TB) and drug-resistant tuberculosis in the last few decades. Although it has been brought under control in most Western countries, it is still a major cause of death in endemic regions like India. Osteoarticular tuberculosis (OA TB) forms a small proportion of the total cases of tuberculosis. Perceptions and practices of orthopedic surgeons are entirely different in endemic and non-endemic regions around the world, due to the vast difference in exposure. Literature from endemic areas puts stress on clinico-radiological diagnosis and empirical anti-tubercular treatment (ATT). Such practices, although non-invasive, simple to implement, and economical, carry a significant risk of missing TB mimics and developing drug resistance. However, OA TB is still perceived as a “diagnostic enigma” in non-endemic regions, leading to a delay in diagnosis. Hence, a high index of suspicion, especially in a high-risk population, is needed to improve the diagnosis. Evolving drug resistance continues to thwart efforts to control the disease globally. This review article discusses the perceptions and practices in different parts of the world, with India as an example of the endemic world, and lays down priorities for overcoming the challenges of diagnosing osteoarticular TB.

## 1. Introduction

Tuberculosis (TB) is one of the oldest recognized diseases of mankind with significant morbidity and mortality [1]. It is interesting to know that six decades ago, the war on TB was considered “winnable with tools at hand” [2]. However, because of its adaptable nature and help from its partner in crime, i.e., HIV, TB has not only bounced back but has also metamorphosed into drug-resistant TB [3,4]. Multidrug resistance (MDR) is defined as resistance to at least both isoniazid and rifampicin. Extensive drug resistance (XDR) is defined as resistance to any fluoroquinolone, and at least one of three second-line injectable drugs (capreomycin, kanamycin, and amikacin), in addition to multidrug resistance [5]. Despite the Herculean efforts from the World Health Organization (WHO) and multiple national and international organizations to curb the prevalence, 10 million infections were reported globally in 2018.

However, the disease load is not uniform, with a greater prevalence in low-income countries around the world. As per the WHO, the 2018 figures are about 220 per 100,000 in African and Southeast Asian countries, whereas they are less than 30 in the United States and Europe [6]. Unfortunately, some of the high-income countries are witnessing an increase in the incidence of TB largely attributed to massive migration from disease-endemic regions [7].

Osteoarticular tuberculosis (OA TB) represents between 1% and 3% of the TB population, with variable incidence in endemic and non-endemic areas of the world. This has resulted in varying perceptions and practices in these different areas. With high load and poor infrastructure, surgeons in India have adopted the practice of initiating empirical anti-tubercular treatment (ATT) based on clinical and imaging findings. This concept of “clinico-radiological” diagnosis of OA TB in endemic areas is based on a presumably better clinical acumen due to high patient load, the easy availability of imaging modalities like magnetic resonance imaging (MRI), the shortage of laboratory facilities, and slow culture and drug sensitivity results. However, such practices increase the risk of missing TB mimics and drug-resistant TB. In contrast, surgeons in high-income countries often do not initiate investigations for mycobacterial disease, leading to a delay in diagnosis, and hence consider this as a “difficult clinical diagnosis” and a “diagnostic enigma” [8].

Thus, the priority is to improve practices throughout the world, helping to institute a “protocol-based treatment” in endemic areas and improving “perceptions” in non-endemic areas.

### 1.1. Epidemiology with India as an Example of the Endemic World

In 2018, an estimated 10 million people globally became infected with TB with almost half a million new cases of resistance, of which 78% were MDR TB. India bears almost 27% of the global burden of new cases as well as resistant cases. Additionally, every year, a million “missing” undiagnosed or inadequately diagnosed cases are not notified [9]. In 2018, the reports of the first national anti-tuberculosis drug resistance survey by the Ministry of Health and Family Welfare Government of India (GOI), 2014-16, showed that 23% of new TB patients had mono-resistance and 3% were MDR. This was the first nationwide survey done in India [10]. These high numbers have left world leaders in the healthcare sector extremely concerned.

The true incidence of OA TB is unknown and there are no epidemiological data or studies available. We can, however, only extrapolate the incidence representing 1–3% of pulmonary TB. OA TB appears to follow the trends of pulmonary TB, and a high incidence of resistance is reported in the study by Kapil et al. In a series of 686 culture-positive cases of Koch’s spine studied at a tertiary referral center, drug resistance was noted in 111 (16%) cases with 87 (12.7%) being MDR [11]. Primary resistance is also reported in OA and extrapulmonary TB by other authors from India [12,13].

### 1.2. Epidemiology in the Non-Endemic World

The European Union (EU) and European Economic Area (EEA) as well as the United Kingdom (UK) comprise 31 countries with a population of 517 million. The report “Tuberculosis surveillance and monitoring in Europe 2020 (2018 data)” states that, in 2018 in the EU/EEA and the UK, there were 52,862 tuberculosis patients, with a notification rate of 10.2 per 100,000 population. Of all notified TB cases, 40,625 (76.9%) were newly diagnosed and 36,047 (68.2%) were confirmed by culture or smear and nucleic acid amplification test. Of all TB cases with reported HIV status, 4.2% were co-infected with the virus. TB in prisons remains poorly reported; for the 17 EU/EEA countries reporting data, the notification rate amounted to 217 new and relapse TB cases per 100,000 inmates, that is, a relative risk of 14.7 compared to the general population in the same countries.

Thirty-five per cent of all TB cases reported in the EU/EEA were of foreign origin, but a recent analysis failed to demonstrate a positive correlation between migration and incidence of TB in Europe [14]. Of 26,881 cases with the relevant drug susceptibility test (DST) results, 999 (3.7%) had MDR TB. The highest percentages (more than 10%) of MDR TB cases were reported from Estonia and Lithuania. XDR TB was reported for 19.6% of 808 MDR TB cases tested for second-line drug susceptibility. The rate of notified MDR TB cases decreased from 0.3 per 100,000 population between 2014 and 2016 to 0.2 in 2017 and remained the same in 2018 [15]. The overall notification rate and the rates in most EEA countries have been decreasing over the last five years [15].

A recent Centers for Disease Control and Prevention (CDC) report from the United States shows a decrease in incidence from 9 cases per 100,000 persons in 1993 to 2.8 cases per 100,000 persons [16].

The incidence of OA TB in non-endemic areas is unknown. OA TB should be part of differential diagnosis with a high index of suspicion in the prison population, people with foreign travel, any unusual presentation, and patients suffering from HIV.

## 2. Etiopathogenesis

OA TB occurs primarily by hematogenous spread from a primary focus such as lung, kidney, and lymph node. OA TB is often a reactivation of an OA lesion that has been implanted at the time of initial bacteremia [17].

**Figure 1:**
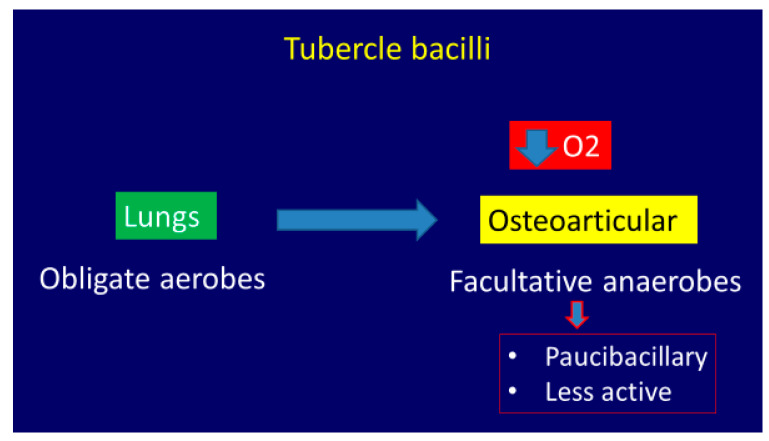
The pathogenesis of osteoarticular tuberculosis (OA TB).

Tubercle bacilli are obligate aerobes in the lungs but become facultative anaerobes in situations when there is reduced oxygen tension. Thus, bacilli are rather less active in the osteoarticular lesions than the pulmonary ones (paucibacillary) (Figure 1) [18].

More than 50% of OA TB affects the spine. Thoracic vertebrae are the most commonly affected region (50%) [19]. Hematogenous spread of the infection in the paradiscal regions occurs from the vascular plexus in the subchondral region of each vertebra and the intra-osseous venous systems or the Batson’s paravertebral venous plexus.

In the appendicular skeleton, the bacteria reach the joint space directly through the sub-synovial vessels or indirectly by eroding the epiphyseal bone. In the appendicular skeleton, the hip and the knee are the most common sites affected [20].

### 2.1. Clinical Features

Skeletal tuberculosis can affect any gender or age group in the endemic regions, but it is mostly seen during the first three decades of life and equally in both men and women [19].

**Figure 2:**
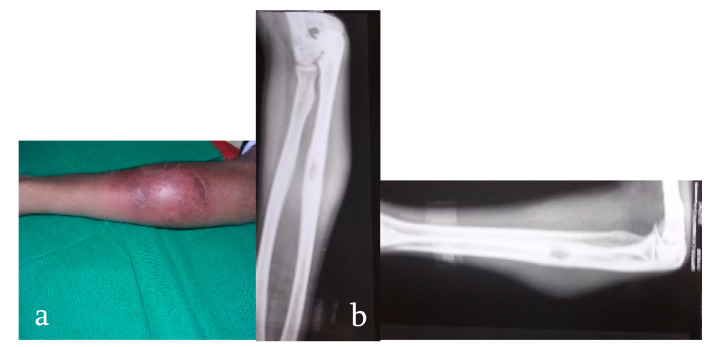
(**a**) An 18-year-old female presents with a gradually progressive soft to firm swelling over her mid-upper forearm for 2 months, suggestive of a cold abscess. (**b**) X-rays suggestive of lytic lesion in the diaphysis of the ulna with significant soft tissue swelling around it, suggestive of infective pathology. Final diagnosis: pan-sensitive TB.

Patients generally present with joint pain, deformity, and the characteristic cold abscess, sinus, or a tubercular ulcer. Classical constitutional symptoms associated with tuberculosis like low-grade evening fever, anorexia, and weight loss are seen in less than one-third of patients and an active concomitant pulmonary infection is rarely seen [21]. On examination, muscle wasting, localized tenderness, regional enlarged and matted lymph nodes, and painful restricted adjacent joint movements are commonly seen. A cold abscess can become subcutaneous locally (Figure 2) or penetrate ligaments, bone, and periosteum and travel along fascial planes and neurovascular bundles like cervical triangles, along the chest wall and iliac fossa [20].

**Figure 3:**
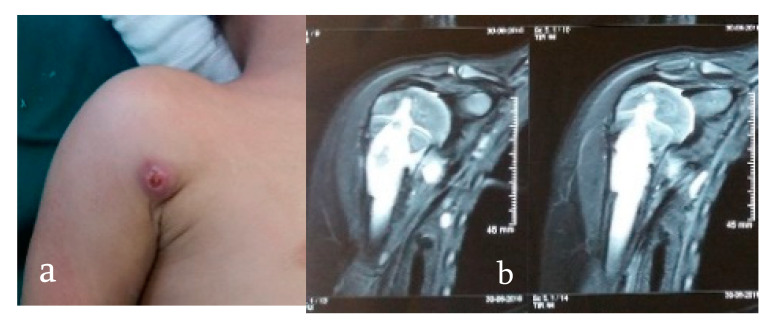
(**a**) An 11-month-old boy presents with discharging sinus over right anterior axillary fold with restricted and painful movement of his right shoulder. (**b**) Magnetic resonance imaging (MRI) reveals infective lesion crossing the physeal plate of the proximal humerus.

The tubercular sinus has a wide mouth and thin, blue undermined margins with serosanguinous discharge. The sinus has a thick wall and can be fixed to the underlying tissue or bone (Figure 3) [22].

**Figure 4:**
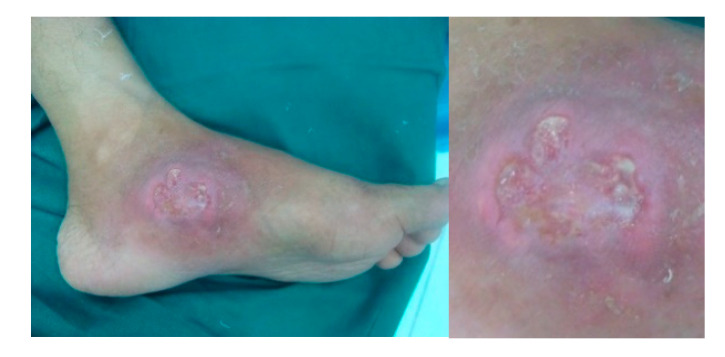
A characteristic tuberculous ulcer in the foot of a 60-year-old male. It is characterized by thin, reddish-blue undermined edges. There is pale granulation tissue with scanty serosanguinous discharge in the floor and slight induration at the base.

The tubercular ulcer is slightly painful. It has thin, reddish-blue undermined edges. There is pale granulation tissue with scanty serosanguinous discharge in the floor and slight induration at the base (Figure 4) [22].

The disease most commonly involves the osteoarticular region but may rarely present as osteomyelitis.

The osteoarticular disease goes through five stages in its natural history [23].

1. Stage of synovitis. Patient presents with night pain and spasm of the affected group of muscles. Affected joint has >75% of the joint movement preserved. X-ray shows rarefaction and haziness of the articular regions. This stage has excellent prognosis.
2. Stage of early arthritis. Patient presents with joint pain, spasm of the surrounding muscle, and difficulty in doing some activities of daily living. Affected joint has 50–75% of the joint movement preserved. X-ray shows classical Phemister’s triad of juxta-articular osteopenia, mild joint space reduction, and peripheral osseous erosions. This stage has good prognosis with mild stiffness.
3. Stage of late arthritis. Patient presents with joint pain, spasm, and difficulty in activities of daily living. Affected joint has >75% loss of range of movement of the joint. X-ray shows marked joint space reduction and joint destruction. This stage has fair prognosis with severe loss of motion.
4. Stage of advanced arthritis with subluxation and dislocation. Patient presents with deformity of the involved joint. Affected joint has gross restriction of all movements. X-ray shows pathological dislocation with wandering acetabulum in the hip. This stage has poor prognosis.
5. Terminal or sequelae of arthritis. Fibrous ankylosis of the joint is seen.

### 2.2. Imaging Features

X-rays: The commonly observed features on X-rays in spinal TB are loss of vertebral height, indistinct vertebral endplates, erosions, angular kyphosis, and paravertebral masses. Involvement of two or more contiguous vertebrae generally occurs later [24].

**Figure 5:**
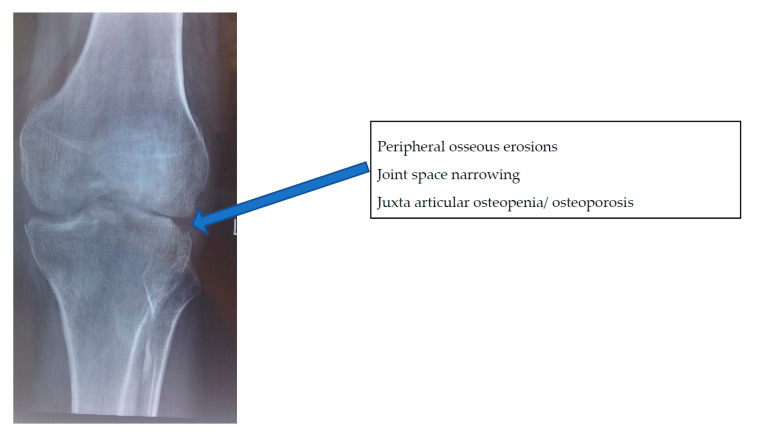
The characteristic Phemister’s triad of peripheral osseous erosions, joint space narrowing, and juxta-articular osteopenia/osteoporosis in a case of tuberculosis of the knee in a 30-year-old man.

In appendicular skeleton, Phemister’s triad of juxta-articular osteopenia/osteoporosis, peripheral osseous erosions, and gradual joint space narrowing suggests tuberculous arthritis but is nonspecific (Figure 5).

OA TB can rarely present as tuberculous osteomyelitis involving long bones. When it involves flat bones of the hand or foot, medullary expansion is seen by the classic picture of “spina ventosa”.

The MRI is very sensitive with respect to bone and soft tissue changes.

**Figure 6:**
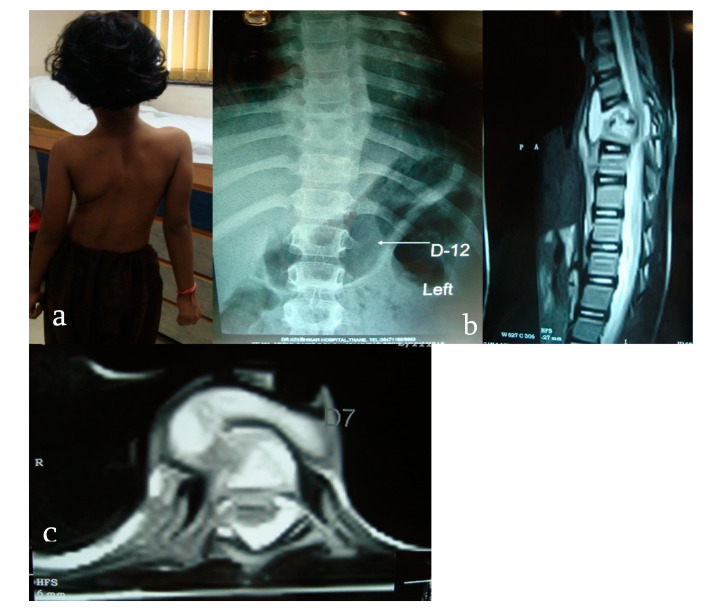
(**a**) A 6-year-old girl presents with scoliosis noticed by parents 4 months previously. (**b**) X-ray of the dorsal spine showing collapse of the D8 vertebra and a fusiform-shaped soft tissue shadow suggestive of an abscess. (**c**) MRI of the dorsal spine showing collapse of the D8 vertebral body with marrow edema, prevertebral and paravertebral collection with subligamentous and epidural extension, and epidural involvement (canal encroachment).

MRI findings considered to be diagnostic of tuberculosis of the spine are a combination of marrow edema, paravertebral collection with subligamentous and epidural extension, contiguous vertebral body involvement with relative preservation of disc space, epidural involvement (canal encroachment), intraosseous abscess, and septate paravertebral shadows (Figure 6) [25].

Cord edema, myelomalacia, cord atrophy, and syringomyelia are observed in cases with neural complications. Healing or healed disease is suggested by resolution of marrow edema and collection, fatty replacement of bone marrow, resolution of cord signal intensity, and absence of contrast enhancement [26].

**Figure 7:**
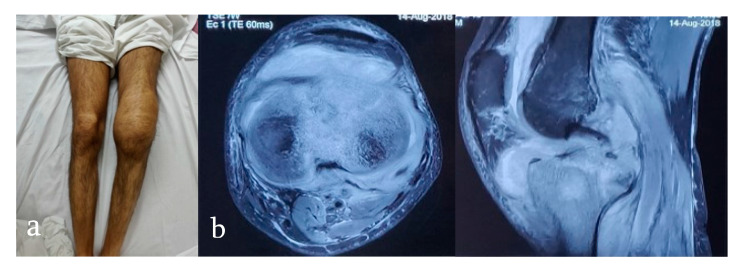
(**a**) A 28-year-old man presents with pain, gradually progressive swelling in his left knee and restricted left knee movements since the past 3 months. (**b**) MRI left knee shows synovitis, bone marrow edema with erosions, osteomyelitis with para-articular abscess, and tenosynovitis.

In the appendicular skeleton, the most common MRI findings are synovitis, bone marrow edema and erosions, osteomyelitis with para-articular abscess, and tenosynovitis (Figure 7) [21,27]. However, MRI findings are sensitive but not specific.

The CT scan is a valuable tool when the X-rays are normal and MRI facilities are unavailable. It is particularly useful for skeletal locations not clearly visible on radiographs like craniovertebral spine, cervicodorsal spine, rib, sternum, sacroiliac joint, and posterior elements of vertebrae. The CT scan detects abscess within the vertebral canal. Varying degrees of bone destruction with presence of sequestrum is pathognomonic of TB on a CT scan [28]. Presence of sequestrum, osteosclerosis, and epidural or soft tissue abscess are an important aspect of diagnosis [28,29]. Dystrophic calcification is well determined by CT scan and calcification of an abscess is pathognomonic of TB [30]. A CT-guided biopsy is one of the most common modes to biopsy a lesion for microbiological and histopathological investigation.

Positron emission tomography/computed tomography (PET/CT) imaging allows three-dimensional localization of tracer activity to bone lesions and is useful for differentiating soft tissue infection from bone infections. It is used to assess sites of suspicious bone infection and can observe the extent of the lesion at complex anatomical sites [31]. Fluorodeoxyglucose (FDG)-PET/CT could be the ideal investigation to decide the end point of treatment [32,33].

## 3. Diagnosis of Osteoarticular Tuberculosis

### 3.1. Obtaining an Appropriate Sample for Laboratory Studies

An appropriate sample must be obtained for processing when OA TB or any OA infection is suspected.

Cotton swab specimens from discharging sinuses should be avoided. These specimens are unreliable for isolation of mycobacteria or they may contain skin contaminants skewing culture reports [34].

Tissue samples yield the best results [35,36]. Tissue can be obtained by biopsy under image guidance or by arthroscopic procedures or during open surgical procedures.

While some authors favor fine-needle aspiration cytology in superficial affected joints [37], some do not recommend it and favor wide bore or J needle [38].

Percutaneous transpedicular biopsy is favored by most spine surgeons for obtaining a sample of suspected spinal tuberculosis [39].

Arthroscopy provides visualization of the lesion, biopsy as well as option for surgical excision if needed.

The samples (pus and tissue) for bacteriological examination are collected in a predesignated sterile leak proof container and transferred to the laboratory at the earliest to process them for pyogenic and mycobacterial smear and culture. The histopathological sample must be collected in a formalin container [40].

### 3.2. Culture, Genotype Diagnosis, and Drug Susceptibility

TB culture is still considered as the gold standard in the diagnosis of tuberculosis. Additionally, it also helps us in assessing drug resistance.

The commonly used culture media are as follows:

(A) Lowenstein–Jensen (LJ) medium is the conventional medium. It is a solid medium requiring basic infrastructure with low cost and is highly specific for *Mycobacterium tuberculosis* (*M. tb*). However, it has low sensitivity especially in smear-negative pulmonary TB and extrapulmonary TB. Additionally, reports are available at six to eight weeks and delay in laboratory diagnosis of TB is a major obstacle in TB control programs.

(B) BACTEC Mycobacterium Growth Indicator Tube 960 (MGIT 960) is a liquid medium and is sensitive, accurate, and reproducible. It has a better yield and the culture report results are available within two to three weeks as opposed to the six to eight weeks needed for LJ medium [41,42,43,44]. MGIT detects *Mycobacterium tuberculosis* (*M. tb*) complex consisting of *M. tuberculosis, M. bovis, M. africanum and M. microti*. However, it is more expensive and its implementation requires an advanced technical infrastructure that is not always available in resource-poor countries [45].

Laboratory technicians require special training in safe practices for specimen and culture handling. Risk of contamination or carryover contamination exists because of the use of a highly enriched growth media for culture [46].

In short, MGIT is the top performer with regard to sensitivity, while LJ medium sets the current standard for specificity. Hence, international guidelines recommend that all specimens that are cultured on automated liquid systems be inoculated on solid medium as well [47].

(C) Bilayered Medium for Rapid Isolation of *Mycobacterium tuberculosis*: This medium consists of a lower layer of LJ medium and an upper layer of Middlebrook (MB) 7H10 with an added indicator to detect growth by color change. The isolation rate is almost double and it has a better recovery time than that of LJ, and it is less expensive than MGIT. However, its role in OA TB has not yet been proven.

Drug sensitivity studies: These should always be done whenever mycobacterial culture is positive. The importance of performing drug sensitivity studies can be judged when one reviews the literature on drug-resistant cases of OA TB from endemic areas. It reveals any one drug resistance to be around 30% and MDR to be between 9% and 25.5% (Table 1).

Culture is the gold standard and is the only method to assess complete drug sensitivity; however, deep inaccessible lesions, paucibacillary state, and initiation of empirical ATT pose difficulties in the accurate diagnosis of OA TB [50]. Most Indian studies have reported poor culture yield (10% to 30%) in OA TB [21,51]. Even a very large study carried out over six years at a major reference laboratory from India reported positive yield in only 189 out of 1295 extrapulmonary specimens with MGIT culture [52].

Molecular Methods: These methods can detect *M. tb* as well as resistance.

GeneXpert is a novel test used to detect the presence of *Mycobacterium tuberculosis* DNA with results available within two hours. Moreover, it can detect rifampicin (RIF) resistance, helping to start therapy immediately for MDR TB. The specificity of GeneXpert is 100% and the sensitivity is quite low for OA tuberculosis (about 60% to 70%). A negative GeneXpert cannot be used to rule out tuberculosis infection and, thus, culture remains the gold standard [53].

A new diagnostic tool, GeneXpert MTB/RIF Ultra or Xpert Ultra, has improved sensitivity in the detection of TB and RIF resistance compared to GeneXpert and line probe assays (LPAs) in both pulmonary and extrapulmonary samples, but at the cost of lower specificity than the GeneXpert [54,55]. GeneXpert Ultra is negative in non-tubercular mycobacteria (NTM), a close TB mimic, and thus helps to differentiate them in smear positive (AFB smear) cases. This is particularly important as the incidence of NTM infections is increasing in some parts of the world [56,57].

Pyrosequencing is a rapid method for the detection of resistance to rifampicin, isoniazid, streptomycin, ethambutol, ofloxacin, and amikacin in *M. tuberculosis*. It is useful as a molecular diagnostic tool for screening and predicting resistance to re-treatment of pulmonary TB patients [58]. The results are available within six hours but the primary limitation is the cost of the test [58].

**Figure 8:**
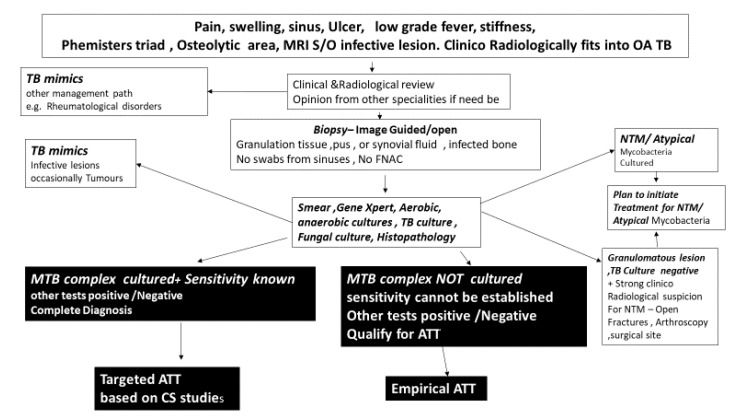
A simplified management algorithm to follow in suspected cases of OA TB [41,80].

The management flowchart for a suspected case of osteoarticular tuberculosis is presented in Figure 8.

Drug sensitivity studies with molecular methods: It must be borne in mind that most current molecular methods detect rifampicin resistance, where a few others detect resistance to isoniazid. Resistance to other drugs may go unnoticed if only these methods are used, and the patient may receive one or two ineffective drugs [59].

Hence, these methods must be supported by culture-based drug sensitivity tests.

### 3.3. Smear and Histopathology

Smear: Ziehl–Neelsen (ZN) stain is a rapid method to detect acid-fast bacilli (AFB). The number of bacilli needed for detection are considered to be 10,000 per slide or ml, and hence sensitivity is often low especially in biological fluids (5% to 20%) [60]. The sensitivity can be increased by using Auramine O (AO) fluorescent dye and by concentrating and combining these two methods of staining. However, staining cannot differentiate between *M. Tb* infection and non-TB mycobacterial infection [61].

Histopathology: The microscopic pathological lesion of TB is the “tubercle” characterized by central necrosis surrounded by epithelioid cells, giant cells, and mononuclear cells. Two types of lesions have been described in the literature: (a) caseating exudative type—more commonly seen in children characterized by rapid and extensive destruction of bone and cartilage with predominating caseation and cold abscess formation; and (b) proliferating type—seen in adults mostly characterized by less bone destruction and cellular proliferation predominating with minimal caseation. The tuberculous granuloma is the extreme form of the caseating type [62]. The specimen for histopathology in formalin container should be sent in all cases of biopsy and debridement [63,64,65,66].

### 3.4. Indirect Diagnosis—Serological Tests

Although many tests have been introduced for the indirect diagnosis of TB, serodiagnosis has limited utility in disease-endemic countries with high infection rates and an acquired immunodeficiency syndrome (AIDS)-prevalent population [67]. Interferon-gamma release assays (IGRAs) are not sensitive or specific enough to diagnose extrapulmonary TB, especially in endemic countries with a high disease burden [68,69]. Still, they are often used as an attractive alternative to invasive methods of diagnosing TB. However, it may be noted that poor performance of all commercial serodiagnostics, consequent misdiagnosis, and wastage of resources have prompted the WHO to issue a disapproval [41].

### 3.5. Clinico-Radiological Diagnosis

This is the most common method of diagnosing OA TB in India, and is based on clinical and imaging features without attempting biopsy, and hence is prone to miss TB mimics and drug-resistant cases [70,71,72]. However, when all the tests are inconclusive, the clinic-radiological diagnosis remains the only approach to initiate ATT. This situation is common in endemic as well as developed and non-endemic countries [73,74].

Thus “astute clinicians and comprehensive diagnostic algorithms” play a vital role in clinico-radiological diagnosis [75]. However, it must be understood that, although it is popularly called clinico-radiological “diagnosis”, there are no clinical and/or radiological criteria to diagnose OA TB (unlike rheumatoid arthritis). Hence, at best it can be called clinico-radiological impression acting as a threshold for initiation of ATT.

**Figure 9:**
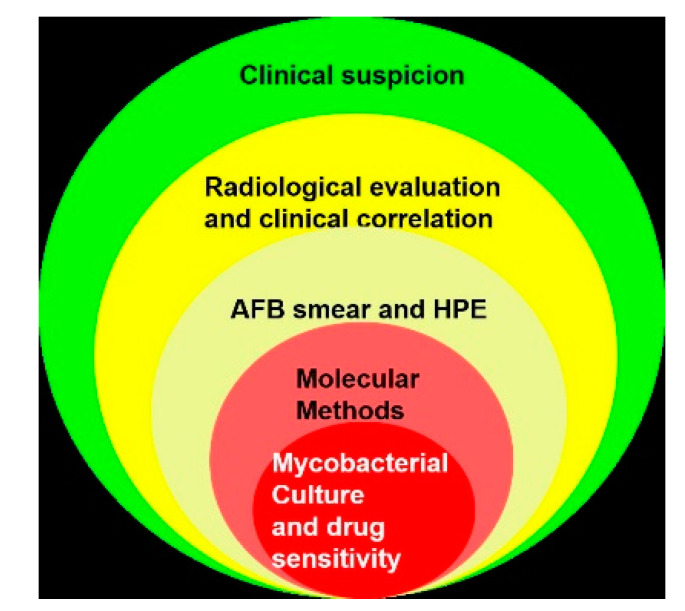
The hierarchy of investigative modalities for the diagnosis of TB. AFB, acid-fast bacilli; HPE, histopathological examination.

To summarize, there is no diagnostic test which can diagnose OA TB with high sensitivity and specificity. The hierarchy of investigative modalities for diagnosis of OA TB. is shown in Figure 9.

### 3.6. Differential Diagnosis

1. Non-tuberculous mycobacteria (NTM). Initiating ATT would not yield desirable results in cases of NTM [71].
2. Pyogenic arthritis
3. Rheumatoid arthritis (RA). In RA, the disease is usually polyarticular, the synovium tends to be thicker and irregular, and erosions are smaller as compared to OA TB [76].
4. Pyogenic spondylodiscitis. Presents as single segment involvement with poor soft tissue mass and early disc changes on MRI [77].
5. Seronegative spondyloarthropathy, especially while dealing with sacroiliac lesions.

The other diagnoses to be entertained are tumors and pigmented villonodular synovitis in the appendicular skeleton, herniated disc, canal stenosis, and mechanical back pain in cases of spinal TB [78,79].

## 4. Discussion

The world TB scenario improved dramatically in the middle of the 20th century when anti-TB drugs were introduced to the world. Dr. B. Mukhopadhaya from Patna, India, while delivering the Hunterian lecture on OA TB at the Royal College of Surgeons in 1955, had concluded saying that we appear to be on the “threshold of success” [81]. However, today, we appear to be on the “threshold of failure” in pulmonary as well as OA TB in an endemic country like India, with a high number of new cases, increased drug resistance, and increased mortality in both an HIV as well as non-HIV population.

India reported totally drug-resistant TB (TDR-TB) in 2012 shortly after Iran [82,83]. Although the title was challenged later, it represented a very serious situation. There were several possible reasons for this trend in a country like India. The onset of TDR-TB could be linked to an HIV epidemic that at the turn of the century hit Indian metropolises in a big way. In a paper aptly entitled “TB and HIV, partners in crime”, Maniar et al. described the situation in Mumbai between 1999 and 2003. They studied 8680 HIV-positive patients in Mumbai, 93.5% of whom were co-infected with TB, of which 42% culture-positive patients were drug resistant [4]. Naturally, they acted like epicenters of the drug-resistant TB. Megacities like Mumbai became a hotspot for TB and HIV because of high population density, malnutrition, and pollution with inadequate water and sanitation facilities, providing the ideal environment for the spread of TB, the failure of treatment, and the emergence of resistance [84].

The irrational prescription of ATT by family physicians could have been another cause of rising resistance. A study done among family physicians serving Dharavi in Mumbai, the largest slum in Asia and a TB hotspot, revealed that only 6 out of 106 physicians could write an appropriate prescription for first-line ATT and these 106 doctors prescribed 63 different regimens for TB [85]. Poverty and the inability to afford ATT resulted in improperly administered ATT, and the inability to adhere to treatment regimens contributed to drug resistance in low-income countries.

The current situation is described by Chatterjee et al. in an eye-opening article entitled “Drug-resistant tuberculosis: is India ready for the challenge?”. The authors mentioned that resistance to fluoroquinolones is 21% in non-MDR and 36% in MDR patients and resistance to aminoglycosides such as amikacin, capreomycin, or kanamycin is 7% in new patients. In addition, resistance to ethionamide and para-aminosalicylic acid (PAS) was 11% in new patients [73].

**Figure 10:**
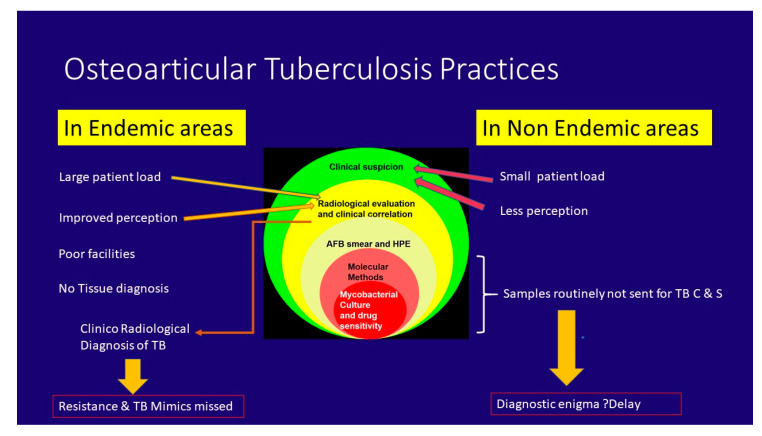
OA TB practices in endemic and non-endemic areas of the world.

This increased resistance in pulmonary TB naturally translated into increased resistance in OA TB, and this has been noted in a few studies from India mainly about metropolitan cities [11,41]. Such assessments were not done in smaller cities or in villages. The reasons are many; first and foremost is the lack of affordable access to reliable drug sensitivity testing centers. As of 2017, there were only 143 centers for universal drug sensitivity testing in a vast country with more than 700 districts and a population of more than 1.3 billon [58]. In addition, reportedly poor culture yield in OA TB and unaffordability by patients have resulted in orthopedic surgeons’ preference of initiating empirical ATT based on clinico-radiological findings. Hence, the exact incidence and nature of resistance remain masked (Figure 10).

At the other end of the spectrum, OA TB is quite rare in developed countries. The low incidence can be judged by the fact that, in a 20-year study of OA TB in children from a pediatric center in Spain, there were only 11 cases of OA TB. Of these, 9 patients were children of immigrants [74].

Once a probable diagnosis of OA TB is made, the cases may be investigated along the lines discussed below:

1) Ruling out false-positive and false-negative cases and achieving probable diagnosis of TB.
  a) Confirming that other conditions are not treated as tuberculosis (Ruling out false positive);
  b) Establishing that TB is not treated as other conditions (Ruling out false negative).
2) Achieving accurate diagnosis of TB and establishing sensitivity pattern, ideally in every patient of OA tuberculosis.
3) Performing diagnostics in patients who were treated with empirical ATT and not responding to ATT.
  a) Ruling out false-positive cases: Empirical ATT is often erroneously initiated in NTM infections and pyogenic infections, among many others.
    NTM infections are often not even considered and ATT is instituted when histopathology reveals granulomatous infections. Some details of history like infection following open fractures or invasive procedures like arthroscopy or intra-articular injections would point to NTM infection [71]. It is best to inform the microbiologist about the suspicion so that appropriate tests are carried out. Another important feature in NTM infections that helps in early diagnosis is a positive smear for acid-fast bacilli and negative GeneXpert [75,76,77,78,79,80]. The incidence of these infections is sizeable. In an Indian study of 1295 extrapulmonary TB suspects, NTM was grown in 72 specimens, while *M. tb* was cultured in 189 specimens [51].
    Pyogenic infections are to be ruled out in every case of suspected OA TB by smear and culture for pyogenic organisms. In a study of 93 patients referred from city public hospitals and private healthcare facilities across Mumbai on the threshold of receiving ATT for suspected OA TB, 7 out of 93 were diagnosed to be suffering from pyogenic infections [41]. Other conditions such as rheumatoid arthritis and ankylosing spondylitis can be differentiated from OA TB by a good clinical and radiological examination and an appropriate referral to a rheumatologist.
  b) Ruling out false negative: Confirming that TB is not treated as other conditions. Several studies from non-endemic areas or developed countries have reported a delay in diagnosis and treatment for OA TB, mainly because the condition is not high on the clinician’s differential diagnosis list. The medical community should bear in mind that immigration from disease-endemic regions has reintroduced the relatively rare tuberculosis infection.
    In a paper entitled “Delays in the diagnosis and treatment of bone and joint tuberculosis in the United Kingdom”, Broderick et al. described a median delay of seven months between the onset of symptoms and the referral to an appropriate center in 30 patients who were finally diagnosed as OA TB; 89% of their patients were migrants. They also noted that in 23% of the patients the initial sample was not sent for mycobacterial culture at the time of index surgery at the tertiary center, needing a second surgery [86].
    Arrabal et al. described a three-year delay in diagnosis of TB of the hip joint in a 54-year-old patient in Spain, even though he had multiple discharging sinuses around the hip. Based on a culture from the sinus that grew coagulase-negative *Staphylococcus aureus*, he was initially treated with targeted antibiotics [87].
    Thus, it must be borne in mind that there is an increased incidence of this “difficult clinical diagnosis” in developed countries [8] (Figure 10).
4) Establishing a sensitivity pattern ideally in every patient of OA tuberculosis: The importance of establishing a sensitivity pattern can be judged by a recent publication on prevalence and patterns of drug-resistant pulmonary tuberculosis in India by Lohiya et al. [58]. They found the incidence of any one drug resistance at 24.9%, MDR at 3.5%, and XDR at 0.6% in new cases. INH (isoniazid) resistance was highest at 16% while rifampicin was at 4.7%, suggesting that rifampicin sensitivity cannot be considered as a surrogate marker of INH sensitivity. In re-treatment cases, the incidence of MDR was 26.7% and any one drug resistance was 58.4%.
  Since OA TB is likely to follow the same trend, targeted ATT is essential to addressing the challenge of drug-resistant OA TB infection. Prerequisite to this is improving the yield of the *M. tb* culture. Appropriate sample and container, early transfer to a laboratory with experience to tackle extrapulmonary specimens, and availability of liquid culture medium play an important role in improving the yield of the sample.
5) Diagnosing and Addressing Non-Respondents: After the initiation of empirical ATT based on either clinico-radiological or histopathological findings, the patients must be closely observed.
  If a satisfactory response is not determined in about three months, a close review is needed to find the reason for poor response. The poor response can be judged by persistent or increased swelling, development of new swelling or new lesion, persistent discharging sinus, development of new sinus, development of non-healing ulcer, neurological deterioration, or development of new neurological deficit. Agashe et al. under the aegis of the Bombay Orthopaedic Society studied 89 patients not responding to ATT at a mean duration of 9.32 months (3 months to 60 months). Surprisingly, 80.9% patients were initiated on ATT without tissue diagnosis. Of the 89 patients, 33 had resistance to at least one drug, 24 were MDR, and 3 were XDR. Noteworthy findings were varying resistance pattern, primary resistance to second-line drugs, and high number of resistant patients in the pediatric age group. On comparing the resistance pattern with another Bombay Orthopaedic Society project conducted between 2004 and 2007, the authors noted significant deterioration in the resistance pattern [41]. Another concerning observation was that 16/89 patients were suffering from TB mimics. The authors urged for a drastic change in mindset in diagnosing and managing OA TB in India and have stressed that it is not adequate to only diagnose OA TB but establishing a drug sensitivity pattern is important [38].

This drastic change in the management of OA TB in endemic regions like India would mean a significant improvement in infrastructure, a change in mindset of orthopedic surgeons and doctors at large, and the willingness on the part of patients to undergo invasive investigations for a condition “traditionally” treated with medicines empirically.

Similarly, a drastic change would be needed in the mindset of surgeons in non-endemic regions to put OA TB on a “suspect list”.

To summarize, with the epidemic of drug resistance in a paucibacterial disease like OA TB, the definitions must be modified. We propose them as follows:

1. “Complete diagnosis of OA TB”—i.e., culture-positive cases with drug sensitivity known and in whom targeted chemotherapy can be initiated.
2. “Qualify for ATT”—i.e., ATT is initiated based only on clinical and imaging findings or on results of tests that do not establish a sensitivity pattern. This is an acceptable practice but only after attempts at establishing a “complete diagnosis” have failed and the surgeon strongly suspects OA TB.
3. The term “clinico-radiological diagnosis” should be replaced by clinico-radiological impression as there is no consensus on clinical and/or radiological criteria for diagnosing OA TB.

Protocol-based therapy is very difficult, particularly in cash-strapped economies, but even in progressive economies like India (6th largest GDP in the world) it would be tricky to implement it. In India, there are currently 628 GeneXpert machines and 74 National Tuberculosis Elimination Program (NTEP)-certified laboratories to perform susceptibility testing [10]. These facilities against a load of 2.8 million new cases and 150,000 cases of MDR per year appear grossly inadequate.

If current practices are not modified in India, it is postulated that, by 2032, the incidence of MDR may increase by 275% and an estimated 85% of these cases will be primary resistant tuberculosis [88].

Tremendous political will is needed at the national level to tackle such a deteriorating scenario. Significantly more resources, up from the current 1.28% of GDP (2017–18) that India spends on public healthcare, are needed to improve poor infrastructure [89]. The number of laboratories must be increased urgently. The community education program especially related to hygiene and close contact must be tackled locally but supported on a national and international platform.

Associations and societies in endemic areas, such as the Indian Orthopaedic Association (IOA), must take rapid steps to educate its members on coming out of their comfort zone of instantaneous diagnosis obtained by non-invasive means and trying to achieve “complete diagnosis”.

Similarly, orthopedic societies in developed countries must educate their surgeons on keeping OA TB in mind when dealing with a case of osteoarticular infection.

The WHO will have to funnel its resources to increase and upgrade laboratories in developing nations and endemic areas.

Lastly, international bodies such as the World Association Against Infection in Orthopaedics and Trauma (WAIOT) and the SICOT (Société Internationale de Chirurgie Orthopédique et de Traumatologie) can play a major role in research by connecting endemic and non-endemic countries. The high volume of patients in endemic areas and the advanced technology available in developed and non-endemic countries can help us understand more about this disease.

## 5. Conclusions

1. TB is a tenacious disease having bounced back from the brink of defeat to a position of infecting 10 million people annually.
2. The incidence of TB is disproportionately high in India, China, and some African countries.
3. Drug resistance is increasing in endemic areas and, at present, at least a quarter of new TB cases are resistant to at least one drug and about 3% have MDR. Resistance to fluroquinolones, aminoglycosides, ethionamide, and PAS are also noted in new cases.
4. OA TB is paucibacterial, often slowly progressive, accompanied by constitutional symptoms in about one-third to one-half of cases. Spinal TB is the most common presentation of OA TB.
5. OA TB constitutes between 1% and 3% of the TB population and, hence, surgeons in endemic areas are exposed to a greater patient load of OA TB cases as compared to surgeons in non-endemic areas.
6. Surgeons in endemic areas tend to “over-diagnose” TB as tissue diagnosis is hardly obtained due to the lack of infrastructure, a reportedly poor culture yield, the confidence in clinical acumen, and a belief in MRI. The initiation of empirical ATT is almost a norm in most parts of India.
7. While surgeons in non-endemic areas often “under-diagnose” OA TB because pyogenic infections are always high on the list and investigations are directed accordingly, this often leads to a delay in diagnosis.
8. The initiation of ATT is a fateful decision; the practice of empirical ATT, although reasonably successful in previous years, is certainly not advisable in view of rising drug resistance. Every attempt must be made to establish a drug sensitivity pattern before the initiation of ATT.
9. Due to the paucibacterial nature and moderate culture yield at best, empirical ATT must be started in a proportion of cases even if the culture yield is negative, if OA TB is strongly suspected. These patients must be observed very carefully and, if a good response is not obtained after three to four months, the cause for the poor response must be investigated and addressed.
10. Infrastructure development on a priority basis is the need of the hour in endemic regions and an improved perception is required in non-endemic regions.

## Figures and Tables

**Table 1:** TB culture and drug sensitivity in patients with OA TB from endemic areas.

Author, Country, Year	Culture-Positive TB	Number of TB-Resistant Cases (MDR, Multidrug Resistance; XDR, Extensive Drug Resistance)
Li L et al., 2012 [48]	127/249	Any one drug resistance—39 (30.7%); Of these, MDR—12/127 (9.5%)
Xu L et al., 2013 [49]	76/152	Any one drug resistance—23/76 (30.1%); MDR—16/76 (21%)
Agashe et al., 2009 [41]	47/74	Any one drug resistance—5/47 (32%) MDR—12/47 (25.5%); XDR—2/47 (4.3%)
Mohan K et al., 2013 [11]	686	Any one drug resistance—111/686 (16.2%); MDR—87/686 (12.7%); XDR—3/686 (0.4%)
